# Alexander Luria and Jean Piaget: Partial Reconstruction of Their Cooperation

**DOI:** 10.1002/jhbs.70033

**Published:** 2025-08-20

**Authors:** Marc Ratcliff, René van der Veer

**Affiliations:** ^1^ Centre Jean Piaget, Faculty of Psychology and Educational Sciences University of Geneva Geneva Switzerland; ^2^ Institute of Education and Child Studies Leiden University Leiden Netherlands

## Abstract

In this paper the genesis of the cooperation between Alexander Luria and Jean Piaget is reconstructed on the basis of recently found correspondence and historical data. It is shown that their initial meeting at the famous Ninth International Congress of Psychology in New Haven, 1929, formed the beginning of a cordial and productive relationship which, however, was abruptly interrupted in the late 1930's. At the background of much of their cooperation was the monumental figure of Lev Vygotsky, whose work Luria attempted to promote in the West and who for Piaget, in a way, would probably become the most valuable interlocutor he never met.

## 1 Introduction

Shortly after the publication of Vygotsky's *Thought and language* (Vygotsky 1962), the MIT Press published a booklet with a comment of 14 pages by Jean Piaget (cf. Burman et al. 2025). In that comment Piaget (1962, 1) wrote “It is not without sadness that an author discovers, twenty‐five years after its publication, the work of a colleague who has died in the meantime, when that work contains so many points of immediate interest to him which should have been discussed personally and in detail. Although my friend A. Luria kept me up to date concerning Vygotsky's sympathetic and yet critical position with respect to my work, I was never able to read his writings or to meet him in person, and in reading his book today, I regret this profoundly, for we could have come to an understanding on a number of points”. Piaget then went on to discuss Vygotsky's criticisms of his viewpoints on egocentrism, egocentric speech, and conceptual development.

This comment was highly interesting, because it suggested that Piaget was aware of Vygotsky's criticism of his work, although perhaps not in sufficient detail to react to it in writing, and that it was Vygotsky's immediate collaborator Alexander Luria who kept him informed. Indeed, it is quite possible that Piaget acquainted himself with the general flavor of Vygotsky's critique and the tenets of cultural‐historical theory through his frequent contacts with Luria which developed from 1929 onwards. First, Vygotsky's and Luria's view on Piaget's concept of egocentric speech was presented by Luria at the Ninth International Congress of Psychology in New Haven in 1929, where Piaget also gave a talk. Second, at that congress Luria and Piaget developed a friendship and presumably discussed each other's views in detail during their frequent meetings. Third, after the congress Luria and Piaget began corresponding about scientific issues, possibly about Vygotsky's ideas (see above). Fourth, Luria was instrumental in publishing Piaget's first two books in Russian and asked Piaget to contribute a preface to the Russian edition (Piaget 1932c), which also contained a lengthy critical introduction by Vygotsky (Vygotsky 1932). Fifth, after the publication of that volume Luria and Piaget kept corresponding until 1936, when historic events interrupted their contact. In that correspondence, Luria asked Piaget, among other things, to join one of his psychological expeditions to the Caucasus and to contribute a chapter to the Festschrift which he began compiling after Vygotsky's death in 1934. Finally, in the mid 1950s Luria and Piaget renewed contacts and met each other during Piaget's visits to Moscow in 1955 and 1966 (Garcia et al. 2025). In this paper, we will try to reconstruct these contacts between Luria and Piaget on the basis of the historical literature and recently discovered documents and correspondence. It will be shown that both Luria and Piaget were active networkers who believed in and promoted international scientific cooperation. They greatly respected each other's works and tried to maintain a constructive cooperation under difficult circumstances and against the background of the tensions between the Soviet Union and the West. By retracing the genesis of their relation we shall also show Luria's role of a mediator between Vygotsky and Piaget, who unfortunately never met each other. On the one hand, he was instrumental in promoting Piaget's ideas in Russia and, on the other hand, he actively promoted Vygotsky's work in the West with Piaget's help. In the late 1930s sociopolitical events caused the contacts between Luria and Piaget to be severed and it took 20 years before they were resumed. It would be Piaget who after World War II played a diplomatic role in restoring the relationship between Soviet and Western psychology and who facilitated the spread of Vygotsky's ideas by writing an introduction to the translation of his main work.

## 2 Luria's and Piaget's Early Career

When Luria and Piaget first met at the Ninth International Congress of Psychology they were not exactly newcomers in the field. As a student, Luria became interested in psychoanalysis and at the age of twenty he founded a psychoanalytic society in his native town Kazan. He immediately notified Freud and began sending brief reports of the society's activities to the *Internationale Zeitschrift für Psychoanalyse* (e.g., Luria 1922). After his move to the Psychological Institute of Moscow University in 1923, where he obtained the position of associate professor (“scientific collaborator of the first category”), Luria also joined the Russian Psychoanalytical Society as its secretary. He gave talks at their meetings, analyzed patients, published reports and theoretical papers (Luria 1925, 1926), wrote a preface for the Russian edition of Freud's *Beyond the pleasure principle* (Vygotsky and Luria 1925), and took care of the society's organizational matters and international correspondence. At the Psychological Institute and at the psychology laboratory of the Academy of Communist Education, where Luria got the position of director of the psychology section, his research first focused on issues that derived from his psychoanalytic interest, e.g., the measurement of (hidden) emotions (Luria 1928a, 1928b; Luria and Leont'ev 1926). After 1924, however, Luria began cooperating with Vygotsky and his interests gradually broadened to include child development (Luria 1929a), cross‐cultural psychology, and psychopathology (Mecacci 2024). The context of much of the research into child development was the reform of the Russian educational system, which required innovative curricula based on a thorough knowledge of child development (Van der Veer 2020). With Vygotsky, Luria and his students replicated many of the findings of the leading Western researchers, such as Köhler, Piaget, and Stern, and often offered alternative explanations. Around 1927, the theoretical background of their activities became the theory of cultural development elaborated by Vygotsky and Luria, which essentially stated that human development is characterized by the appropriation of cultural tools (Luria 1928c; Vygotsky 1928a, 1928b, 1929). Luria and Vygotsky believed that this theory could be part of a new psychology based on Marxist principles and regularly criticized Western, “bourgeois,” theories (Luria 1928d). More than Vygotsky, Luria developed and maintained contacts with foreign colleagues. In July 1925, he first visited the Institute of Psychology in Berlin and became acquainted with Kurt Lewin and his team of largely female students of whom at least five were of Russian descent (Gita Birenbaum, Tamara Dembo, Nina Kaulina, Maria Ovsiankina, and Blyuma Zeigarnik) (Woodward 2010). Luria also became a member of the editorial board of the *Journal of Genetic Psychology* in 1928, published in foreign journals and corresponded with international researchers and publishers.

Nevertheless, it seems fair to say that up to 1929 Luria was relatively unknown in the West (see also the remark by Langfeld below). This cannot be said of Piaget, who was something of a rising star in the late 1920s, a specific period in his thinking (Ducret 2011; Montangero and Maurice‐Naville 1997; Beilin 1992) marked by a model for explaining mental development that appeals to social mechanisms, in particular socialization and cooperation (Kitchener 2009). Piaget joined the Rousseau Institute in 1921 as head of the psychology laboratory, where he found the ideal environment for developing a new research culture (Ratcliff 2024). This combined simplicity of experimentation with the complex art of the clinical method, enabling him to investigate the processes of knowledge formation in children in an unprecedented way. From 1921 to 1925, Piaget and his team of 25 female and two male collaborators produced a vast collection of data (Mayer 2005; Bond and Tryphon 2009; Morelli and de Souza 2020). It was on the basis of these data, to which must be added the data collected in Paris between January 1920 and May 1921 (Morelli Ribeiro 2018), that Piaget wrote his first four books between 1923 and 1927. The first two (Piaget 1923, 1924) focused on the child's logic, with particular emphasis on language and reasoning, while the next two (Piaget 1926, 1927) concerned the child's explanations of the world. In 1925, Piaget was appointed to a professorship at the University of his native Neuchâtel (Perret‐Clermont and Barrelet 2008). From there, in 1926, he launched a new research program on moral judgment in children, while continuing to collaborate with the Rousseau Institute. But the atmosphere in Neuchâtel, which did not allow his research culture to take root, did not suit him, as he admitted in a letter to his friend Ignace Meyerson (“Neuchâtel is becoming increasingly unlivable”, Piaget 1928a).^1^ So, as early as 1927, Piaget began to devise a strategy for returning to Geneva, even if it meant accepting a lower‐level position than that of ordinary professor he held in Neuchâtel (Ratcliff and Borella 2013). And indeed, in 1929 he was appointed extraordinary professor in Geneva.

With four books and dozens of articles, Piaget's publishing record in this period was impressive but it would be wrong to think that he stayed put between Geneva and Neuchâtel. In fact, Piaget was a regular traveler, having developed a culture of naturalist travel in his teens, as part of his early training as a malacologist, which he continued on study trips to Zurich and Paris from 1918 to 1921 (Ducret 1990). In Switzerland, between 1921 and 1929, he gave lectures, sometimes several times, in Geneva, but also in other cities such as Ste‐Croix, Neuchâtel, La Chaux‐de‐Fonds, Berne, Fleurier, Brugg, Rolle, La Sarraz, Davos, Lucerne and Lausanne. Outside Switzerland, Piaget visited Berlin (1922), Cambridge (1927), Liège (1928), London (1927), Madrid (1929), Paris (1923, 1925, 1927, 1928, 1929), Thônon (1922), and The Hague (1929). It's clear, then, that he left no stone unturned in a classic academic career in which networking is important, if not essential.

Piaget's publications and lectures did not go unnoticed and he was soon considered a classic author (Wallon 1927). His method and findings greatly stimulated the interest in child psychology and a lively debate about his interpretations ensued. Psychologists, philosophers, medical doctors, and pedagogues criticized several of his central ideas: the notion of childhood egocentrism or autism, the overly strong influence on development allegedly accorded to the social factor, the maturational approach, and the notion of the impermeability of the child's mentality. In various countries, colleagues tried to replicate Piaget's findings (e.g., Bourjade 1927; Isaacs 1930; Katz and Katz 1928; Stern 1927) with mixed results and Piaget replied to their criticisms (Parrat‐Dayan 1993a; b). It was in this well‐established context that Piaget traveled to New Haven and first met Alexander Luria.

## 3 The Presentations in New Haven

Luria gave two presentations at the congress, one about the measurement of emotional reactions, which continued his research inspired by psychoanalysis, and the other about egocentric speech, based on replications of Piaget's work. The first paper, presented in the evening session on 3 September, outlined Luria's new objective method to study ‘affective traces’ in criminals and ‘hysterics’, which he would elaborately test with Aleksey Leontiev and Viktor Lebedinskiy (cf. Luria 1929b) and present several years later in his book *The nature of human conflicts* (Luria 1932). Subjects were asked to hold their finger on a pneumatic appliance while verbally responding to the experimenter's questions. The disturbances of the motor curve expressed the corresponding affective change evoked by the questions, Luria (1930a, 295) claimed. Attempts to conceal affective reactions were not successful and ‘innocent’ subjects did not show the specific motor reactions. Thus, what Luria presented was in essence a contribution to reliable lie detection (Trovillo 1939, 114). The second paper, presented in the morning session on 4 September, dealt with Vygotsky's and Luria's (1930b) ideas about the function and fate of egocentric speech and thus must have attracted the attention of Piaget. This presentation showed an unexplored dimension of egocentrism, that is, its possible role as a precursor of inner speech. Luria told his audience that Vygotsky and his collaborators in a series of experiments had discovered the psychological mechanisms responsible for the evolution of egocentric speech and its function. In his words, interrupting children's activity elicited a flurry of egocentric speech, which evidently was directed towards the solution of the problem and addressed social others. Luria argued that children first stated the problem verbally to organize their subsequent activity. Thus, egocentric speech had a problem‐solving function and did not simply reflect the child's general autistic attitude as Piaget had allegedly claimed (In fact, Piaget (1928b) had already abandoned the autistic model to replace it by sensori‐motor intelligence). Moreover, Luria claimed that structural resemblances suggest that egocentric speech does not simply disappear but turns into inner speech and retains the function of organizing the child's behavior. In sum, Vygotsky and Luria had supposedly discovered the function and fate of egocentric speech, which thus forms an intermediate stage between external social speech and internal private speech.

Piaget's presentation took also place in the morning session on 4 September and dealt with the alleged parallelism between the development of logic and moral reasoning in the child. He thus reported on the field of moral development that he had been studying since 1926, linking it to logical development, which he had studied a few years earlier. Egocentrism, which “is not an unsocial behavior but a state of absence of differentiation between the social and the purely individual” (Piaget 1930d, 339), requires the action of certain social processes to be overcome and leave place to logic. He presented the two social mechanisms of constraint and cooperation, whose effect on egocentrism is either to help it become established or to eliminate it. Piaget argued that the evolution of logic parallels the evolution of morality, where egocentrism can be strengthened by unilateral respect and diminished by cooperation.

## 4 The 1929 Congress

The Ninth International Congress of Psychology at Yale University, New Haven, was a major event that attracted more than one thousand visitors, among whom were many of the most eminent psychologists of that time (Boring 1929; Cattell 1929; Duncan 1980; Langfeld 1929; Poffenberger 1929; Sutherland 1929). The Soviet delegation, consisting of Vladimir Borovskiy, Solomon Gellershteyn, Isaak Spil'reyn, and Alexander Luria, all collaborators at the Moscow Institute of Experimental Psychology (Van der Veer and Valsiner 1991, 128–131), arrived in New York on August 23, about a week before the actual congress started (Luria 1930b, 82; Yasnitsky 2012). The presence of the Soviet delegation didn't go unnoticed. Sutherland (1929, 305) remarked that “Soviet Russia was well represented at the Congress, and one was glad to hear something, at any rate, trustworthy about Russia. Psychological studies are being actively pursued and one gathered that, in spite of his immense authority, there are other points of view in Russian psychology than that of professor Pavlov, and that the psychologists of the State Institute for Experimental Psychology protest against his complete mechanization of human behavior.” Langfeld (1929, 366), in his account of the congress, noticed the presence of “A.R. Luria, of Moscow, prominent among the younger group of Russia and a specialist in educational psychology”.

The Swiss delegation consisted of six people (five according to the proceedings; cf. Boring 1930, 4), notably Edouard Claparède, Piaget, two female students of the Rousseau Institute, the Lausanne professor and coeditor with Claparède of the *Archives de Psychologie*, Jean Larguier des Bancels, and a Bernese, Walter Heller. In essence, most of them were part of the Institute's network. The other Swiss school of psychology – Zurich –, with a psychoanalytic and psychiatric orientation, had not sent any members. Of the six participants, only Claparède and Piaget gave lectures.

### 4.1 Precongress Program

The early arrival of the Soviet psychologists gave Luria and his colleagues the opportunity to participate in the pre‐congress program for the approximately 200 foreign visitors. Among them, Germany was particularly well represented with Gustav Kafka, David Katz, Kurt Koffka, Wolfgang Köhler, Kurt Lewin, Otto Lippmann, Hans Rupp, and Wilhelm Stern. Other Europeans included Karl and Charlotte Bühler, Raymond Cattell, Edouard Claparède, Ovide Decroly, Anton Grünbaum, Albert Michotte, Henri Piéron, Jean Piaget, and Charles Spearman. On Friday morning, August 30, the foreign psychologists took a train to Princeton University, where Herbert Langfeld, director of the Psychological Laboratory, explained the ongoing research in his lab. After lunch, Howard Warren, head of the Psychology Department, gave them a guided tour at the campus. On return to New York, the visitors were met at the railway station by representatives of Columbia University, conveyed to the campus and assigned rooms at Furnald Hall (Sutherland 1929). The next morning, Saturday August 31, visits were made to, among other things, the Colombia Medical Center and the Psychiatric Department of Bellevue Hospital. In the afternoon there was a sight‐seeing bus trip around New York and in the evening an excursion to the giant Roxy cinema. Finally, on Sunday, September 1, the guests were transported to Yale University in New Haven and accommodated in the Harkness Memorial Quadrangle. This busy pre‐congress program gave the foreign psychologists ample opportunity to get acquainted with American psychology but also, and perhaps even more importantly, to get to know each other and become befriended. As Langfeld (1929, 366) noted, “the fact that all the psychologists lived in the same building helped to preserve the unity of the unusually large group, and the beautiful court of the dormitory offered an inviting place for many impromptu discussions, which frequently lasted until the small hours of the morning”.

### 4.2 Congress Program

The congress itself began on Monday, 2 September, under terribly hot conditions: it was 94 degrees Fahrenheit (34.4℃) and James McKeen Cattell began his presidential address by inviting the attendants to take off their coats (Sutherland 1929, 302). Because of the large number of presentations, there were three parallel morning sessions with six papers plus discussion of each 20 min. In the afternoon, there were five to seven parallel symposia with papers of 8 min, followed by discussion. Finally, each evening saw two longer lectures by distinguished members, notably Pavlov, Köhler, Michotte, Piéron, Lashley, William Stern, Ponzo, Thorndike, and Spearman. By all accounts, it was the 80‐years old Ivan Pavlov, who dropped in from the International Congress of Physiology at Boston held just before the psychological congress, who left an unforgettable impression during his lecture on Monday evening (Duncan 1980; Langfeld 1929; Sutherland 1929). Speaking in Russian and interpreted by his student Gleb von Anrep (Igual 2021), he displayed a remarkable enthusiasm and fervor that electrified the audience. Edna Heidbreder (cited in Duncan 1980, 3) remembers that at one point the empathizing audience broke into enthusiastic applause without waiting for the translation, only to learn a few minutes later that Pavlov had been describing some apparatus in his laboratory. Other lectures that stood out were the one by Thorndike on the role of punishment in learning, Köhler's account of ‘some problems of Gestalt psychology’, and Lashley's talk about the basic neural mechanisms of behavior. Of the Swiss delegation, it was Claparède, who attracted attention. Since the Groningen Congress of 1926 (Boring 1930, 45) he had been the secretary and in New Haven he wrote the minutes of the meeting of the International Congress Committee, chaired by Congress President Cattell (*ibid*., 3–7). He also wrote the minutes of the Business Meeting (7–8) and finally, at the request of the Organizing Committee, presented a history of the International Congresses (*ibid*., 33–47) that followed the welcome words by the President of Yale University and Cattell's address. In his speech—in French—he underlined the fact that this was the first time that the Congress had been held in the USA, a destination regarded as a “promised land” (*ibid*., 33–34), since the congress had been planned to take place there since 1892. He went on to trace the history of the congresses, combining memories and statistical data with the historical argument presented in its international diversity, the evolution of its themes and organization—including a plea for the abolition of individual papers. All the major actors in international psychology were represented, in a unifying and enthusiastic fresco that took into account the differences and specificities of each congress. He underlined the benevolence of psychologists in their post‐war attitude, encouraging meetings after the First World War. Claparède ended his speech with a call for worldwide solidarity, revealing his side as a peace activist.

Some of his prestige was obviously passed on to Piaget, who had just been appointed extraordinary professor of the history of science at the University of Geneva and codirector of the Rousseau Institute. Piaget was Claparède's dauphin and, institutionally speaking, his heir. However, Piaget was handicapped by his poor knowledge of English, which he could read but did not speak at a time when French was still understood by part of the international public.

## 5 Luria‐Piaget Correspondence

Luria's and Piaget's meetings in Yale formed the beginning of an intellectual friendship. From several letters that Luria sent to Kurt Lewin, we know there was a lively interaction between Luria, several Germans, and Piaget (cf. Métraux 2002). On December 12, 1929, for example, Luria sent Lewin a photograph taken during the conference showing Piaget, Katz, and Lewin together (Luria 1929c). Their communication at the conference was, of course, somewhat hampered by language barriers but not to the extent that they couldn't understand each other. Piaget understood English and German and spoke French, while Luria spoke English and German and understood French. It is quite possible that they and other attendants of the conference communicated in a mixture of languages (The congress rules said that presentations had to be given in either English, French, German, or Italian). What we know for sure is that Piaget wrote his subsequent letters to Luria in French and that Luria replied in German. The correspondence they began immediately after the congress was not without its problems: letters and packages sent to and from the Soviet Union tended to disappear in an unpredictable fashion, a situation that lasted until at least the 1980s (cf. Métraux 2002 about Luria's correspondence with Lewin, which suffered from the same problems). Thus, in his letter of February 12, 1930, Piaget wrote to Luria that he was worried to learn that he had not received two preceding letters and that Luria didn't receive a letter he himself sent the previous year. This explains in part why what is preserved of the Luria‐Piaget correspondence is rather fragmentary. All in all, however, Luria and Piaget seem to have managed quite well.

It is clear from the correspondence that Luria left a very favorable impression on Piaget, who was quite open to criticisms of his work in general and seems to have greatly appreciated Vygotsky's and Luria's alternative interpretation of the nature of egocentric speech. In a letter to his friend and colleague Ignace Meyerson written immediately after the congress, Piaget (1929) stated that he met “an amazing guy” (“un type épatant”), who had a “large number of works of first quality” in progress. He also mentioned that Luria “worked with Vygotsky” and suggested that both Luria and Vygotsky should be included in the editorial board of the journal *L'Enfance* Meyerson and he were planning to edit and which never materialized. Also, in February 1930, Piaget wrote to Luria that he had “unforgettable memories of our conversations in Yale” and that Luria's remarks about his research were “the most precious encouragement I have received to date” (Piaget 1930a). Luria replied in May by sending a copy of the recently published *Studies on the history of behavior: Ape, primitive, child* (Vygotsky and Luria 1930a) and in the dedication in French (“To my dear Jean Piaget, with unforgettable memories of our conversations in Yale”) he seems to have copied Piaget's words. That Piaget appreciated Vygotsky's and Luria's input is also clear from the preface to the 1930 second edition of *Language and thought of the child*, in which he mentioned Luria among other authors who criticized his work and announced that he was planning to write a new volume addressing all his critics (Piaget 1930c). Piaget did not mention Vygotsky by name, probably because, in keeping with his philosophy of verification, he wanted to judge by evidence and had no access to Vygotsky's original texts. Probably for the same reason, he included Vygotsky and Luria's early critique voiced in New Haven in a series of criticisms to which he responded in 1933 without mentioning Vygotsky explicitly (Piaget 1933). Indeed, it is quite possible that Piaget never saw one of Vygotsky's relevant texts in print, also because to date no copies of Vygotsky 1934 book or even of the 1932 Russian translation of Piaget's own first works (see below) have been found in the Archives Jean Piaget—now the Jean Piaget Center.

## 6 Piaget's Russian Book

In 1932, Piaget's first two books, *The language and thought of the child*, and *Judgment and reasoning in the child* were published in one volume in the Soviet Union (Piaget 1932b). The Russian edition contained a long critical essay by Vygotsky (1932) and a preface for the Russian reader written by Piaget (1932c). The book was printed in 3000 copies and it is possible that its translator was one of Vygotsky's sisters, Klavdia Semyonovna Vygodskaya (1904–1975), who was a linguist and author of French‐Russian & Russian‐French dictionaries. Vygotsky and Luria had good contacts with Soviet publishing houses and were active promotors of translations of books written by leading Western psychologists, for which Vygotsky often provided a critical yet sympathetic introduction. Thus, they were instrumental in publishing translations of books by, among others, Karl Bühler, Arnold Gesell, Wolfgang Köhler, and Edward Thorndike.

The idea to translate Piaget's seminal books arose around 1929 and it was Luria who asked Piaget for permission to publish them and to write a preface for the Russian reader. Although Piaget already agreed to write the preface in February 1930 (“It goes without saying that I will be happy to provide an introduction to the translation of my first two books,” Piaget 1930a), it would be September 18, 1930, before he finally sent a few introductory pages, with apologies for the delay (Piaget 1930b). In that same letter, he gave Luria permission to publish his next two books as well and congratulated Luria with the completion of his first expedition to the Caucasus, the results of which he was looking forward to hearing about. In his preface for the Russian edition, Piaget (1932c) first thanked “my good friend Alexander Luria” (this became translated as “the Soviet psychologists”) for his efforts to make the translation of his books possible and, above all, for the series of studies he undertook in Russia to “complement and correct” the ones Piaget conducted in Geneva. He then essentially stated that if we wish to know the contribution of individual properties, on the one hand, and the social environment, on the other, to the child's thinking, we need to vary the social environments. In other words, it is not enough to study children in just one social environment, e.g., that of Geneva. Piaget (1932c) conclusion showed how much he valued constructive criticism and replications: “That is why it is a great pleasure for me to have a collaborator and friend as qualified as Alexander Luria, who studies children from social backgrounds very far from mine. Nothing could be more useful to science than this confrontation of the studies of Russian psychologists with the studies done in other countries.”

The small editorial changes in Piaget's original text may seem trivial but they performed a function in the Soviet discourse about foreign science. Replacing “my good friend Alexander Luria” twice by “Soviet psychologists” suggested Piaget's cooperation with Soviet psychology at large (which did not exist at the time), diminished Luria's role in the editorial process, and avoided the notion of friendship with a “bourgeois” scientist.

## 7 The Psychological Expeditions

In 1931 and 1932 Luria published brief reports in international journals about a psychological expedition he had undertaken with a team of Soviet colleagues in several Asian regions of the Soviet Union in the Summer of 1931 (1931a; 1931b; 1932b). The idea was “to investigate the variations in thought and other psychological processes of people living in a very primitive and economic and social environment, and to record those changes which develop as a result of the introduction of higher and more complex forms of economic life and the raising of the general cultural level” (Luria 1931b, 383). This idea followed directly from the theory of cultural development elaborated by Vygotsky and Luria: because higher psychological processes according to this theory depend on the appropriation of cultural tools, it follows that these processes may differ between subjects of different cultures and/or cultural ‘levels’. Luria listed the topics his team had studied and announced another expedition for the Summer of 1932 “with an international character as it is planned to invite foreign psychologists to participate in it” (Luria 1931b, 384). What Luria did not mention was that the 1931 expedition was planned to have an international staffing as well. Already in 1929, he began inviting foreign colleagues to participate in the expedition he and Vygotsky had planned. On December 12, 1929, he wrote to Lewin that they planned an expedition to “the primitive people in Siberia or South‐Asia” for the Summer of 1931 and invited Lewin to participate (Luria 1929c). He added that he believed that his Academy or the Institute for the Ethnology of the East might fund the expedition and that with the support of some “German or American Institute (maybe also Piaget's Bureau)” it would be possible to do really very nice things (“wirklich sehr schöne Dinge”). With “Piaget's Bureau” Luria referred to the International Bureau of Education (IBE) in Geneva, founded by Claparède, Pierre Bovet, and Adolphe Ferrière in 1925 of which Piaget had been appointed director in 1929 (Hofstetter and Schneuwly 2024). The fact that Luria mentioned the IBE as a possible funding agency clearly suggests that Piaget was among those foreign colleagues Luria invited for his first expedition (see below). In the end, however, none of the foreign researchers was able or willing to accept the invitation and the Russian team did not go to Siberia but to Uzbekistan.

Nevertheless, Vygotsky and Luria were quite pleased with the findings of the first expedition (Van der Veer and Valsiner 1991) and immediately planned a follow‐up for the Summer of 1932. Again, Luria invited a score of foreign colleagues. On December 3, 1931, for example, he wrote a letter to Wolfgang Köhler describing the results of the first expedition and inviting him for the second (Luria 1931a). Luria ensured Köhler that they would cover all his costs, invite him for one or two paid lectures, and that a German speaking person (“Dr. Vygotsky or someone from Moscow”) as well as a local assistant would be available to him throughout the trip. He also stated his hope that Koffka, Lewin, and possibly the ethnologist Richard Thurnwald, would participate. What Luria didn't mention in his letter to Köhler is that he had invited Piaget as well, who, however, didn't reply immediately to his invitation. As Piaget explained on April 12, 1932, one the one hand, he was extremely tempted to join the expedition because of all the discoveries it promised but, on the other hand, “I have a 10‐month‐old son and I am working on a large book on the child's first 2 years: there was therefore no question of abandoning my main “Versuchsperson” (“experimental subject”) at such an important moment” (Piaget 1932a). Piaget, who was busy collecting data, was not the only one who declined the invitation and in the end it was just Koffka who would join the second expedition to Uzbekistan (Luria 1934; Van der Veer and Valsiner 1991; Yasnitsky and Van der Veer 2016).

Luria seems to have been disappointed by the limited participation of his foreign colleagues and on March 6, 1932, he wrote to Köhler with a new plan. The second expedition to Uzbekistan he now saw as a preliminary research trip, an intermediate project with as its function to confirm the results of the first expedition and to prepare for a third, much larger, expedition in the Summer of 1933 (Luria 1932c). On March 15, 1932, Luria invited Piaget for this third expedition as well (Luria 1932d) and to this proposal Piaget reacted with enthusiasm: “I accept with great pleasure (…) I will also be happy to take advantage of this opportunity to establish a technical contact between the International Bureau of Education and the services of the People's Commissariat for Public Education (…) I will think about the scientific themes to study during our expedition and will write to you about it as soon as I have ideas” (Piaget 1932a). Unfortunately, the third psychological expedition never took place and Piaget thus missed the opportunity to conduct clinical interviews with children of a non‐Western culture and to meet Vygotsky in person. Moreover, the results of the first two expeditions were published with a delay of 40 years (Luria 1974, 1976). As was explained elsewhere (Van der Veer 2022; Van der Veer and Valsiner 1991), the Russian authorities were not pleased with Luria's conclusion from the first two expeditions that Uzbek subjects were incapable of abstract verbal reasoning, didn't understand metaphorical speech, and tended to define objects in terms of their practical use. A special investigative committee was installed to study Luria's preliminary findings and interview the participants of the expedition and reached the conclusion that Luria's research was pseudoscientific, reactionary, and anti‐Marxist (Van der Veer 2022). This verdict essentially terminated Luria's cross‐cultural research.

## 8 The Vygotsky Festschrift

Soon after Vygotsky's death in June 1934, Luria began preparing a Festschrift for his colleague and friend with the working title *Problems of the development and disintegration of the mind* (Van der Veer 2020). He wrote dozens of letters to his international colleagues and in a letter to Lewin, on September 7, 1936, he stated that he had received contributions by Karl Bühler, Frits Buytendijk, Kurt Goldstein, Kurt Koffka, Kurt Lewin, and Max Wertheimer. Other authors, such as Adhemar Gelb, Karl Lashley, and Piaget were still on his list (Luria 1936c). It remains unclear whether Piaget eventually sent a contribution to the Festschrift. On November 7, 1935; Luria (1935) reminded him that “a year ago you kindly agreed to send us a work for Vygotsky's memorial volume. We are now ready to publish the volume and expect your work—if possible—by January 1‐15”. Piaget, who was extremely busy in this period, did not react and on April 14, 1936, Luria repeated his request: “I wrote to you a few months ago that we are preparing a Vygotsky memorial volume and that it seems very, very desirable to have a work from your pen appear in this volume. I have not received any news from you since then.” Luria added that many psychologists had already sent a contribution (now he mentioned Adhemar Gelb as well) but that they were eagerly awaiting a text by Piaget, preferably about theoretical questions regarding the development of children's thought and behavior. From a letter to Lewin, dated 4 July, 1936 (Luria 1936b), we know that Luria and his colleagues had agreed to submit the proofs of the Festschrift in September at the latest, so it is not surprising that he ended his letter by asking “May I receive your work in April–May?” (Luria 1936a). This time Piaget answered rapidly. Not for the first time in their correspondence, he apologized for the delay in responding, and wrote “I will send you as soon as possible some pages for the volume in memory of Vygotsky. But I am very busy these days and ask you to be patient a little longer” (Piaget 1936).

The Festschrift was never published and to the best of our knowledge its manuscript was never found in the Luria archives, so we do not know whether Piaget eventually sent his promised contribution. But it is significant that Luria apparently held up publication because he wished his friend to submit a chapter. The reasons for not publishing the Festschrift are not entirely clear but we do know that the Soviet sociopolitical changes of the time greatly endangered Luria's position. Vygotsky's and Luria's theory of cultural development became heavily criticized and the results of the first two expeditions elicited fierce attacks (Van der Veer 1991). Moreover, the infamous Pedology Decree of 1936 essentially banned pedology, of which Vygotsky had been one of the most visible figures, as a science (cf. Caroli and Mecacci 2020). To try to publish a Festschrift about this well‐known pedologist may have simply been impossible or unwise. In addition, Luria's own position became quite precarious. The Maxim Gorky Institute of Medical Genetics, where Luria was carrying out twin studies, came under attack because it allegedly defended “Western” genetic theories and rejected Lysenko's ideas about acquired characteristics and unbound plasticity. When Stalin again publicly supported Lysenko in 1935 many geneticists throughout the country were arrested and killed. The Maxim Gorky Institute of Medical Genetics was closed and its director, Solomon Levit, was arrested and executed as an American spy. Under these circumstances, Luria thought it wiser, at the age of thirty‐four, to disappear for a while and to finish his medical studies. As a result, his scheduled talk at the Eleventh International Congress of Psychology in Paris in 1937, which Piaget attended, did not take place (Maller 1938, 69). In fact, Luria fully disappeared from the international scene and stopped corresponding with his international colleagues. It would take twenty years before his Western colleagues heard from him again. To leave international psychology and the Russian capital to become a relatively unknown medical doctor in the provinces may have been a life‐saving step, also because 1936 meant the beginning of Stalin's Great Terror, which resulted in approximately one million deaths (cf. Luria 1994; Van der Veer 2020).

## 9 Resuming Contact After Stalin's Death

The international political developments (e.g., Hitler's rise to power, the civil war in Spain, the annexation of Austria, the Second World War) greatly disrupted international contacts and cooperation in psychology and affected the attendance of the international congresses. Originally planned to be held in Spain in 1936, the Eleventh international Congress of Psychology was moved to France. In his account of that Congress, held in Paris in July 1937; Maller (1938, 69–70) noticed the sharp decrease in the number of German psychologists and suggested suicide and exile as possible causes. The Twelfth Congress of International Psychology, scheduled to take place at Vienna in 1940, was canceled.

After World War II contacts between the Soviet psychologists and their foreign colleagues were not immediately resumed. Soviet psychology and psychiatry were struck by several orchestrated disasters. In 1948, the Jewish Antifascism Committee, founded during the war, was dismantled, its members arrested and killed. This preluded a wave of anti‐Semitism that spread over the country and caused many victims among the intelligentsia. In June 1950, during a joint session of the USSR Academy of Sciences and the USSR Academy of Medical Sciences, the so‐called Pavlovian Session, all diversions from Pavlov's theory were condemned and leading neuroscientists and psychiatrists were fired and arrested. In 1951, Jewish medical doctors were accused of intending to kill party members and government officials—the so‐called Doctors' Plot—and again countless people were dismissed, arrested, and tortured. All this immediately threatened and affected Luria—a Jewish doctor and an opponent of Pavlov's classical conditioning as an all‐embracing principle—and it may have been Stalin's death in March 1953 that saved his life.

The new Soviet regime acknowledged that the Doctors' Plot did not exist and selectively allowed psychologists and psychiatrists to visit foreign congresses again. But now a new political situation affected international cooperation: the Cold War. The Fourteenth International Congress of Psychology, for example, was scheduled to be held in New York but the American Psychological Association asked their Canadian colleagues to host the congress at the expense of the APA. As Conway (2010, 31) noted, “The Americans could not host the conference in New York because of an Act there which prevented known Communists from entering the U.S. and the government feared European Communists might come to be refused admission and embarrass the U.S.” As a result, the congress was held in Montreal. The organizing committee was informed at a late stage that a Soviet delegation of six people would like to attend the congress. This delegation included Vygotsky's and Luria's close colleagues Leont'ev and Zaporozhets, while the other delegates were Boris Teplov and his collaborator Evgeniy Sokolov, Grigoriy Kostyuk of the Research Institute of Psychology in Kiev, and Pavlov's student Ezras Asratyan (Langfeld 1955). Piaget (1956, 343), who was also present in Montreal, mentioned that “he made excellent contact with the Soviet psychologists, especially with Leontiev and Teplov”. Apparently, the idea for Piaget to pay a visit to the Soviet Union arose during their conversations and in 1955 a small delegation of French speaking psychologists arrived in Moscow. It was there that Piaget and Luria resumed contacts after a pause of twenty years. In his account of this visit, Piaget (1956, 343) mentioned that he had been wondering what the present position of certain of his colleagues would be, which was not surprising given Luria's sudden disappearance in 1936.

Luria himself was first allowed to visit a foreign conference in 1957. He attended the Fifteenth International Congress of Psychology in Brussels where he met many of his old acquaintances (e.g., Dembo, Köhler, Ovsiankina, Piaget). He also met Eugenia Hanfmann, with whom he arranged the translation of Vygotsky's *Thinking and Speech* (Luria 1974, 163). It is likely that around this time Luria also first asked Piaget to write a reaction to Vygotsky's critique on his work for that edition. But the story behind the genesis of that heavily abridged book, which would appear as *Thought and Language* in 1962 (see above) merits a separate article. The same is true for the role of Piaget—who by then had been elected President of the International Union of Scientific Psychology—as a diplomat in international psychology at the time (Garcia et al. 2025). Writing an introduction to Vygotsky's book formed part of Piaget's important role as a go‐between in international psychology during the Cold War. Suffice it to say here that Piaget (with Inhelder) renewed the correspondence and scientific cooperation with Russian researchers and would repeatedly visit the Eastern bloc.

The contact between Luria and Piaget also continued (cf. Piaget 1964, 1965, 1966a). Part of their correspondence concerned Piaget's role at the Eighteenth International Congress of Psychology held in Moscow from August 4‐11, 1966. Apparently, Luria had asked Piaget what he would like to present at the congress and Piaget (1964, 1965) replied that he preferred to speak about the theme of interdisciplinarity in the human and social sciences, a theme that was also high on the agenda of UNESCO and about which Piaget would eventually publish (cf. Piaget 1966b, 1973). In addition, Luria wanted to involve Piaget in a symposium on intelligence at the World Psychiatric Association (WPA) Congress, held in Madrid from September 5‐11. For different reasons, Piaget did not attend the congress in Madrid but he played a major role at the congress in Moscow. As he wrote to Luria, he might give an “evening lecture” about the theme of interdisciplinary research, because in his view evening lectures stimulated informal discussions, allowing him to “learn behind the scenes and in conversations what is difficult to acquire through simply reading countless publications” (Piaget 1965). This was important to him, as in future publications he wished to “highlight all possible trends, not just those which I am familiar with” (Piaget 1965). Eventually, at the Moscow Congress, Piaget gave his conference on interdisciplinarity, and, to bring things full circle, began his presentation by thanking the Program Committee and his “old friend Luria” (Piaget 1966b, 242).

In sum, what is left of the correspondence in the 1960s—Luria's letters from this period have not yet been found—suggests that the two researchers resumed their friendly correspondence about possible joint projects despite the political differences between their countries and the general atmosphere of the Cold War.

## 10 Conclusions

In this paper, we partially reconstructed the story of two scientific networkers who tried to transcend borders to establish a productive relationship despite enormous cultural, economic, and sociopolitical differences. After their first enthusiastic conversations at the 1929 New Haven Congress Luria and Piaget began a warm correspondence, which led to several proposals which so far were unknown to historians: Piaget suggested Vygotsky and Luria as members of the editorial board of the journal he and Meyerson planned to edit, Luria arranged the translation of Piaget's first two books into Russian for which Piaget wrote a special preface, Piaget agreed to participate in one of Luria's cross‐cultural expeditions, and, finally, Piaget accepted Luria's invitation to write a chapter for a Festschrift devoted to the memory of Vygotsky. None of these projects was particularly easy to realize and we have seen that several of them failed. Meyerson and Piaget's journal never materialized, Luria's third cross‐cultural expedition with Piaget's planned participation was canceled, and the Vygotsky Festschrift never published.

The projects that Luria and Piaget did bring to completion, notably the translations of Piaget's books into Russian and Vygotsky's book into English, clearly showed the marks of the political situation of the time. The 1962 edition of Vygotsky's book was heavily truncated and stripped of all references to Marx. The changes in Piaget's 1932 book were more subtle. Although Piaget addressed his “good friend Luria” in his preface to the Russian edition, the latter's name was erased and replaced by anonymous “Soviet psychologists.” This suggested that Piaget was familiar with more Soviet psychologists, including Vygotsky, which was not the case. Vygotsky, indeed, appeared only in the shadow of Luria's and Piaget's relationship: he was absent from the New Haven Congress, he had no direct correspondence with Piaget, and his ideas and criticisms were not discussed in the correspondence, at least in the letters that have been preserved. Moreover, the opportunity of meeting Vygotsky in person presented by the Uzbekistan expedition fell through, which meant that Piaget and Vygotsky could not “come to an understanding on a number of points” (see the introduction); and, from the point of view of the history of psychology, this is perhaps the main drama of this story. In addition, it seems that Piaget never received the Russian translation of his books, nor Vygotsky 1934 book. In other words, the story of Piaget and Vygotsky has all the elements of a failed encounter at both the personal and intellectual level, in contrast to the productive relationship between Piaget and Luria which we partially reconstructed in this paper.

## Data Availability

The data that support the findings of this study are available from the corresponding author upon reasonable request.

## Endnotes

1 Copies of all correspondence can be obtained from the first author. Piaget's letters to Meyerson can be found in Archives nationales de France, 521AP/57. Piaget's original letters to Luria and his preface to the Russian book are in the possession of Elena Radkovskaya, daughter of Luria's sister, who kindly supplied copies to the second author. Except for the preface, this material corresponds to the minutes of Piaget's letters from IBE‐UNESCO cited below. Luria's letters to Kurt Lewin were first deciphered and published by Alexandre Métraux (2002) to which we refer. Subsequently, the correspondence was transferred to the Cummings Center for the History of Psychology of the Archives of the History of American Psychology (AHAP), where it can be found in boxes M944‐M949, M1286, and M2931‐M2939. (https://archives.uakron.edu/repositories/4/resources/17) Luria's letters to Wolfgang Köhler can be found in the Köhler Archive of the American Philosophical Society in Philadelphia. (https://as.amphilsoc.org/repositories/2/archival_objects/321532) The correspondence between Luria and Piaget can be found in: 1. the Archives of the IBE Unesco, 186_Correspondance‐344, Personal correspondence of Jean Piaget (I. ‐ M.); 2. The Archives Institut Jean‐Jacques Rousseau, AIJJR_FG_F_7_13_AR Luria.
